# Prevalence trend and burden of foodborne trematodiasis in China from 1990 to 2021 and its predictions until 2030: a comparative study with Japan and South Korea

**DOI:** 10.3389/fpubh.2025.1504218

**Published:** 2025-02-24

**Authors:** Runzhou Ma, Na Li, Chengming Chen, Jianqiang Lan, Huaibin Guo, Wanxing Zhang

**Affiliations:** ^1^Department of Hepatobiliary Surgery, Hebei General Hospital, Shijiazhuang, China; ^2^Graduate School, Hebei Medical University, Shijiazhuang, China; ^3^Department of Ophthalmology, Tangdu Hospital, The Air Force Military Medical University, Xi’an, China

**Keywords:** foodborne trematodiasis, disease burden, East Asia, temporal trend, projection

## Abstract

**Background:**

Foodborne trematodiasis (FBT) poses a significant public health challenge in East Asia, influenced by local dietary practices and environmental conditions. This study evaluates the prevalence trends and disease burden of FBT in China, Japan, and South Korea from 1990 to 2021, with future burden projections until 2030, to guide targeted prevention strategies and public health resource allocation.

**Methods:**

The study utilized data from the Global Burden of Disease (GBD) 2021, including the absolute prevalence, age-standardized prevalence rate (ASPR), disability-adjusted life years (DALYs), and age-standardized DALY rate (ASDALR). Joinpoint regression analyzed the average annual percentage change (AAPC) and 95% confidence intervals (CI) to track FBT burden trends. A comparative analysis was conducted across different dimensions of the burden of FBT among China, Japan, and South Korea, including age, gender, and temporal trends. Additionally, the Bayesian age-period-cohort (BAPC) model projected future FBT burden trends.

**Results:**

From 1990 to 2021, China showed significant reductions in ASPR (41.65%) and ASDALR (47.44%) of FBT. South Korea also noted a slight decrease, yet both had higher rates than the global average. Japan, conversely, saw a notable increase in FBT burden but with an overall lower burden compared to the global average. Males generally exhibited a higher disease burden than females. Future projections indicate a continued decline or stabilization in China and Japan, with a potential slight increase in South Korea by 2030.

**Conclusion:**

The study reveals contrasting trends in FBT burden among the three East Asian countries, with significant declines in China, a slight decrease in South Korea despite higher-than-global rates, and an increasing but low burden in Japan. These insights are crucial for tailoring public health interventions and allocating resources effectively to combat FBT in the region.

## 1 Introduction

Foodborne trematodiasis (FBT) is a group of zoonotic diseases caused by parasitic worms belonging to the class Trematoda, primarily transmitted through the ingestion of contaminated food or water (1). The life cycle of these parasites involves freshwater snails as the first intermediate hosts and fish, crustaceans, and other aquatic organisms as the second intermediate hosts. Human infection typically occurs by ingesting the second intermediate hosts containing viable larvae or consuming metacercariae clinging to aquatic flora (2). FBT is generally non-self-limiting, and its early and mild stages are often neglected because symptoms are not noticeable. The severity of the disease is directly linked to the chronic infections that result from the trematodes’ persistent survival and reproduction in the body, as well as the potential for severe complications. Clinical manifestations of this infection are diverse and depend on the species of trematode and the stage of infection (3). Symptoms often correspond to the organ where the adult worms are located, with common manifestations including abdominal pain, diarrhea, nausea, vomiting, fever, and allergic reactions. In some cases, the migration of trematodes within the host can cause organ inflammation and damage; for instance, clonorchiasis can lead to liver damage and cholangitis, potentially advancing to cholangiocarcinoma, a severe and often fatal form of bile duct cancer. It is noteworthy that the burden of FBT on public health systems is primarily due to the non-fatal chronic health issues and disabilities it induces, rather than direct mortality rates (4). The epidemiological burden of FBT is substantial, with an estimated 56 million individuals affected globally in 2005, amassing to 665,000 disability-adjusted life years (DALYs) (5). Projections for the 2015–2016 period indicate that approximately 75 million individuals worldwide were affected by FBT infections, with an annual increase of 200,000 cases and totaling 1.1 million DALYs.^1^ Based on available data from the World Health Organization (WHO) as of 2019 for 181 countries, the percentage reduction in FBT DALYs compared with 2015 was 24.9%, reflecting a decrease to nearly 890,000 DALYs. This trend continued into 2021, with just over 890,000 DALYs reported, indicating a slight increase from the 2019 figures (6). Despite its significant impact on public health, FBT is categorized as a neglected tropical disease (NTD) by the WHO (7). Due to limited awareness of FBT among at-risk groups and healthcare providers, along with the absence of effective surveillance systems and precise diagnostic tools, some regions are left without reliable epidemiological data. This lack of awareness and data scarcity can result in a substantial underestimation of the actual burden of FBT (8). This underestimation, in turn, leads to insufficient funding for targeted operational guidance and normative control work, further affecting the effective management and control of these diseases. The prevalence of FBT varies by region, with high endemicity in parts of East Asia, Southeast Asia, and some areas of South America, likely due to specific dietary habits and environmental conditions. China, Japan, and South Korea, as neighboring countries in East Asia, share cultural customs that increase the risk of FBT, such as the consumption of raw fish (4). Despite similar cultural and genetic backgrounds, disparities in population demographics and levels of socio-economic development, lead to varying disease burdens and control measures. China, with its large population and complex geography, is among the countries hardest hit by the prevalence of parasitic diseases globally (9). However, through China’s efforts to raise public awareness about FBT through education, implement control policies to standardize various sectors, and increase financial investment to advance primary healthcare, we hypothesize that the burden of FBT in China will decrease rapidly and steadily over time, reducing the gap with Japan and South Korea. This reduction will not only benefit China’s public health landscape but may also influence the relative prevalence and patterns of FBT among these countries, redefining the regional dynamics of FBT. Therefore, understanding these regional disparities is crucial for targeted public health interventions. The Global Burden of Disease (GBD) study, led by the Institute for Health Metrics and Evaluation (IHME) at the University of Washington, has provided a valuable resource for global health data since the early 1990s (10). Utilizing stringent scientific methodologies, the GBD study provides standardized, comprehensive assessments of global health losses, categorized by age, sex, and geographical location, ensuring the data’s consistency and comparability worldwide (11). According to the latest GBD 2021 data on the prevalence and DALYs of FBT, our aim is to provide a detailed description of the changes in the burden of FBT from a temporal trend, age, and gender perspective, using GBD data from 1990 to 2021. Additionally, we predict the FBT burden for these three countries by 2030. This study aids in understanding the current epidemiological burden of FBT in China, Japan, and South Korea, provides information for public health strategies and the equitable distribution of resources, promotes precise interventions, and assists governments and health departments in optimizing control strategies, crucial for alleviating the burden of this NTD.

## 2 Materials and methods

### 2.1 Data source

The latest GBD 2021 dataset offers the most comprehensive estimates of the global burden for 371 diseases and 88 risk factors across 204 countries and territories, stratified by age and sex. GBD 2021 detailed data sources and methods have been described in previous studies (12–14). Detailed disease data are available from the Global Health Data Exchange (GHDx) website^2^ for download. FBT cases were classified according to the International Classification of Diseases 10th Revision (ICD-10 codes B66-B66.8). The GBD 2021 offers a flowchart and narrative detailing the FBT modeling process, including a comprehensive explanation of the data inclusion and analysis procedures (Supplementary material). The DisMod-MR 2.1 model (a Bayesian mixed-effects meta-regression tool) was applied to produce FBT prevalence estimates by age, sex, and year. We expressed the burden of disease in terms of DALYs. The DALY is a measure of overall disease burden, summing the number of years of life lost (YLL) due to premature mortality and the years lived with disability (YLD) due to clinical manifestations of infection (sequelae), weighted for severity (14). All measures were reported as number counts and age-standardized rates per 100,000 population. This age standardization was based on the World Health Organization’s world standard population structure. The institutional ethics committee granted an exemption for this study, as it did not require approval, given that the data from the 2021 GBD are publicly available. This study is an observational study based on GBD data, adhering to the guidelines for accurate and transparent health assessment reporting. We used the GBD 1990–2021 results to assess the trends in the burden of FBT in China, Japan, South Korea, and globally by extracting data on FBT prevalence, DALYs, age-standardized prevalence rate (ASPR), age-standardized DALY rate (ASDALR), and related 95% uncertainty intervals (UI) from GBD data sources. The demographic data, categorized by age and gender for various countries from 1990 to 2021, can be found in Supplementary Table. In our analysis, the age of patients was divided into the following groups: 1–4, 5–9, 10–14, 15–19, 20–24, 25–29, 30–34, 35–39, 40–44, 45–49, 50–54, 55–59, 60–64, 65–69, 70–74, 75–79, 80–84, 85–89, 90–94, and 95 and above, allowing us to examine the impact of FBT across different age demographics and to identify any significant trends or patterns within these specific age groups.

### 2.2 Statistical analysis

The GBD dataset displays diverse age distributions and demographic characteristics. To overcome differences in age structures, we employed age-standardized rates (ASR) to enable a precise evaluation of the changes in the burden of FBT over time, region, and sex. The calculation formula is as follows:

$ASR=\frac{\sum_{i=1}^{A}a_{i}w_{i}}{\sum_{i=1}^{A}w_{i}}$

A denotes the number of age groups in the reference standard population. In parallel, *a_i_* and *w_i_* represent the age-specific rate and the number of individuals within the *i*-th age group, respectively. To describe the trend of changes in FBT from 1990 to 2021, we used Joinpoint regression software (version 4.9.1.0, National Cancer Institute) to calculate average percent changes (APCs), average annual percent changes (AAPCs), and the corresponding 95% confidence intervals (CIs) for each period (15). When the AAPC was greater or less than 0, the trends were further suggested to increase or decrease accordingly (16). The level of statistical significance for hypothesis testing was set at *p* < 0.05. In addition, we predicted the prevalence and DALYs of FBT in China from 2021 to 2030 based on the Bayesian age-period-cohort (BAPC) model. The BAPC model is built upon the framework of the traditional Generalized Linear Model (GLM) within the Bayesian context. The BAPC model applies a log-linear Poisson model to integrate the multiplicative effects of age, period, and cohort. The formula is expressed as follows:

$\eta_{ij}=\log\left(\lambda_{ij}\right)=\mu +\alpha_{i}+\beta_{j}+\gamma_{k}$

In this formula, *λ_ij_* represents the number of cases (or other count variables under study, such as disease burden-related indicators). *μ* is the intercept, denoting the overall average level. *α_i_* stands for the age effect, where *i* (1 ≤ *i* ≤ *I*) indicates the age group at time *j* (1 ≤ *j* ≤ *J*). *β_j_* is the period effect, and *γ_k_* is the cohort effect. The cohort index *k* is calculated by the formula *k* = *M* (*I* − *i*) + *j*, which depends on the age, period index, as well as the length of the age group and the period interval. For instance, in our study, 5-year age groups and annual data were analyzed, which meant that *M* was 5. It is assumed that the age, period, and cohort effects evolve continuously over time. To generate more accurate posterior probability predictions, a Second-Order Random Walk (RW2) is used to smooth these effects. A significant advantage of the BAPC model is that it utilizes the Integrated Nested Laplace Approximation (INLA) method to approximate the marginal posterior distributions. This approach effectively circumvents the mixing and convergence problems introduced by the Markov Chain Monte Carlo (MCMC) sampling techniques in traditional Bayesian methods while maintaining computational efficiency. Through this means, the BAPC model can handle time series data better and is especially suitable for studies related to long-term disease burden prediction (17). In practical operations, we utilized the “BAPC” and “INLA” packages in R. All data analyses were conducted using the software R (version 4.3.1). The demographic data for the projections were derived from the dataset published on the IHME website. Besides, an INLA approach was performed to generate age-standardized projected rates for sensitivity analysis (18).

## 3 Results

### 3.1 The prevalence and burden of FBT in China from 1990 to 2021 and comparison with Japan and South Korea

The prevalence of FBT exhibited a downward trend from 1990 to 2021 in China. The overall prevalence decreased from 36,621,225 cases (95% UI: 31,239,359–43,237,072) in 1990 to 33,317,223 cases (95% UI: 29,251,039–38,353,602) in 2021, marking a 9.02% reduction. Notably, the ASPR dropped significantly from 3,307.89 (95% UI: 2,845.59–3,875.81) per 100,000 population in 1990 to 1,930.22 (95% UI: 1,700.47–2,240.51) per 100,000 population in 2021, showcasing a 41.65% decrease. In terms of disease burden, the DALYs fell from 938,172 (95% UI: 365,200–1,876,415) in 1990 to 768,297 (95% UI: 383,883–1,367,826) in 2021, reflecting a cumulative reduction of 18.11%. The ASDALR also experienced a substantial decrease, from 84.04 (95% UI: 32.98–168.04) per 100,000 population in 1990 to 44.17 (95% UI: 22.08–79.87) per 100,000 population in 2021, representing a 47.44% decrease. Among the population in Japan, there were 265,865 cases (95% UI: 208,630–331,390) of FBT in 2021, marking a 72.57% increase compared to 154,064 cases (95% UI: 123,323–190,148) in 1990. The ASPR per 100,000 population was 141.83 (95% UI: 114.15–173.29) in 2021, 37.69% higher than in 1990. In 2021, FBT accounted for 3,069 (95% UI: 1,371–5,323) DALYs, a significant increase of 82.9% from the 1,678 (95% UI: 847–2,816) DALYs in 1990. The ASDALR was 1.52 (95% UI: 0.76–2.54) per 100,000 population in 2021, which is 38.18% higher than in 1990. In South Korea, the number of FBT cases was 1,708,313 (95% UI: 1,504,742–1,917,655) in 2021 among its population, a 37.87% increase from the 1,239,079 cases (95% UI: 1,202,763–1,281,065) in 1990. The ASR of prevalence was 2,469.11 (95% UI: 2,215.74–2,758.87) per 100,000 people in 2021, 15.88% lower than the 2,935.19 (95% UI: 2,849.88–3,034.07) in 1990. The DALYs caused by FBT increased from 21,631 (95% UI: 14,625–30,167) in 1990 to 31,373 (95% UI: 20,635–45,258) in 2021, a 45.04% increase. However, the ASDALR per 100,000 people decreased from 50.87 (95% UI: 34.75–70.89) in 1990 to 44.53 (95% UI, 29.12–63) in 2021, a 12.46% decrease (Table 1).

**Table 1:** Prevalence and DALYs of FBT in China, Japan, South Korea, and the world in 1990 and 2021.

Location	Number (95% UI)		Change (%)	ASR per 100,000 (95% UI)		Change (%)
	1990	2021	1990–2021	1990	2021	1990–2021
China						
Prevalence	36,621,225 (31,239,359–43,237,072)	33,317,223 (29,251,039–38,353,602)	−9.02	3,307.89 (2,845.59–3,875.81)	1,930.22 (1,700.47–2,240.51)	−41.65
DALYs	938,172 (365,200–1,876,415)	768,297 (383,883–1,367,826)	−18.11	84.04 (32.98–168.04)	44.17 (22.08–79.87)	−47.44
Japan						
Prevalence	154,064 (123,323–190,148)	265,865 (208,630–331,390)	72.57	103.01 (83.72–126.56)	141.83 (114.15–173.29)	37.69
DALYs	1,678 (847–2,816)	3,069 (1,371–5,323)	82.9	1.10 (0.56–1.8)	1.52 (0.76–2.54)	38.18
South Korea						
Prevalence	1,239,079 (1,202,763–1,281,065)	1,708,313 (1,504,742–1,917,655)	37.87	2,935.19 (2,849.88–3,034.07)	2,469.11 (2,215.74–2,758.87)	−15.88
DALYs	21,631 (14,625–30,167)	31,373 (20,635–45,258)	45.04	50.87 (34.75–70.89)	44.53 (29.12–63)	−12.46
Global						
Prevalence	51,551,241 (46,262,646–58,206,273)	44,466,329 (40,017,218–50,034,921)	−13.74	1,054.95 (948.75–1,188.64)	526.74 (473.70–593.25)	−50.07
DALYs	1,251,259 (610,899–2,236,570)	998,029 (569,766–1,638,112)	−20.24	25.74 (12.58–46.04)	11.79 (6.73–19.46)	−54.2

### 3.2 Joinpoint regression analysis of the burden of FBT in China, Japan, South Korea, and the world from 1990 to 2021

**Figure 1:**
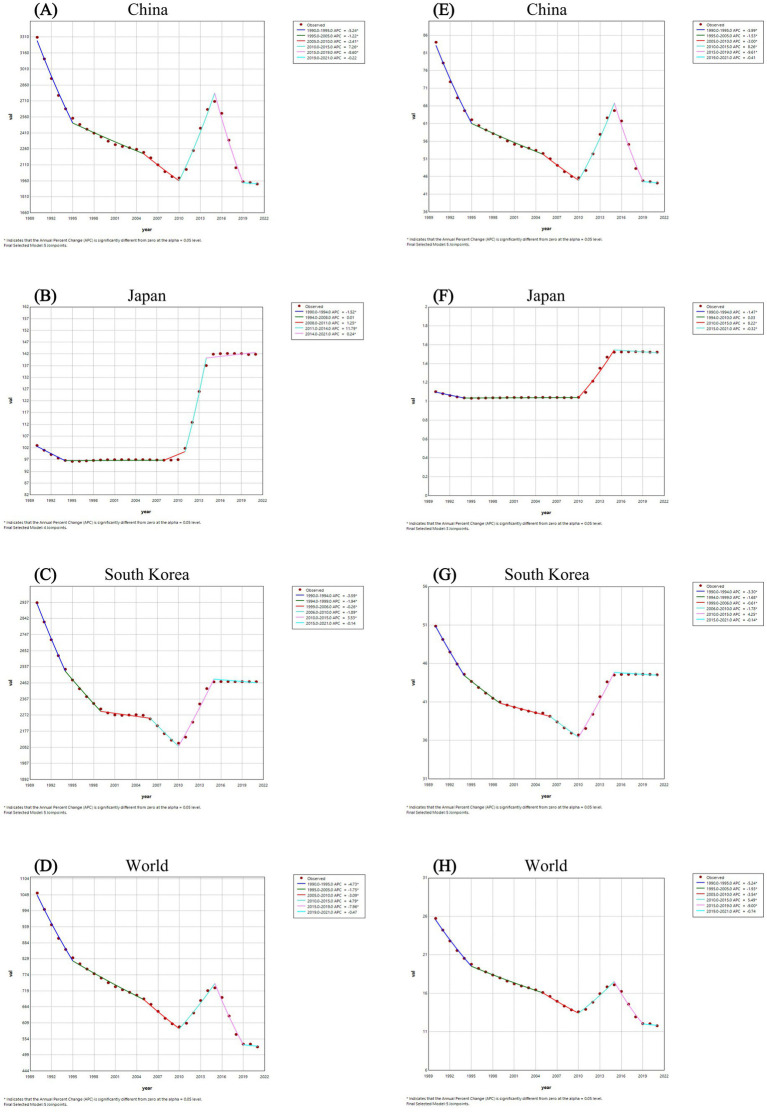
The Joinpoint regression analysis of ASPR for FBT in China **(A)**, Japan **(B)**, South Korea **(C)**, and worldwide **(D)** from 1990 to 2021, and the analysis of ASDALR for FBT in China **(E)**, Japan **(F)**, South Korea **(G)**, and worldwide **(H)** over the same period.

**Figure 2:**
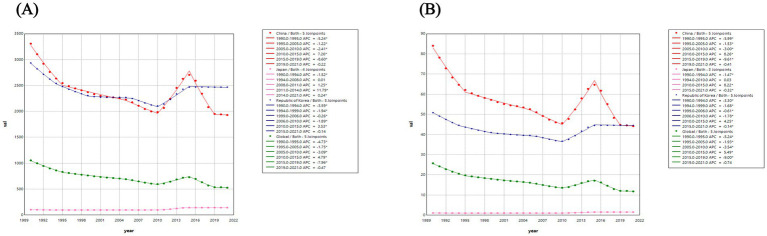
Trend comparison of ASPR **(A)** and ASDALR **(B)** of FBT in China, Japan, South Korea, and worldwide from 1990 to 2021.

The Joinpoint regression analyses of the ASPR and the ASDALR for FBT in China, Japan, South Korea, and worldwide from 1990 to 2021 were depicted in Figures 1, 2. The annual percentage change (APC) for both ASPR and ASDALR in China exhibited significant declines from 1990 to 1995 (ASPR: APC = −5.24; ASDALR: APC = −5.99, *p* < 0.05). There was a subsequent moderate decrease for both rates until 2010 (ASPR: 1995–2005 APC = −1.22; 2005–2010 APC = −2.41; ASDALR: 1995–2005 APC = −1.53; 2005–2010 APC = −3.00, *p* < 0.05). However, there was a slight increase from 2010 to 2015 (ASPR: APC = 7.26; ASDALR: APC = 8.26, *p* < 0.05), followed by a subsequent decline from 2015 to 2019 (ASPR: APC = −8.60; ASDALR: APC = −9.61, *p* < 0.05). The most recent period between 2019 and 2021 remain steady for ASPR and ASDALR. The APC for ASPR in Japan indicated a period of significant increase from 2011 to 2014 (ASPR: APC = 11.79, *p* < 0.05), followed by a stable trend from 2014 to 2021 (ASPR: APC = 0.24, *p* < 0.05). The APC for ASDALR from 2010 to 2015 was similar to that of ASPR (ASDALR: APC = 8.22, *p* < 0.05), with a slight decline from 2015 to 2021 (ASDALR: APC = −0.32, *p* < 0.05). From 1990 to 1994, the APC for ASPR and ASDALR in South Korea showed a significant downward trend (ASPR: APC = −3.59; ASDALR: APC = −3.30, *p* < 0.05). This decline then moderated between 1994 and 1999 (ASPR: APC = −1.94; ASDALR: APC = −1.68, *p* < 0.05). The slope of the trend flattened from 1999 to 2006 (ASPR: APC = −0.26; ASDALR: APC = −0.61, *p* < 0.05). The trend then declined again between 2006 and 2010 (ASPR: APC = −1.89; ASDALR: APC = −1.78, *p* < 0.05). A reversal occurred from 2010 to 2015 (ASPR: APC = 3.53; ASDALR: APC = 4.25, *p* < 0.05), indicating an upward shift in the trend. After that, the APC trend leveled off until 2021.

Meanwhile, the AAPC of FBT prevalence and DALYs in China from 1990 to 2021 was −1.69 (95% CI −1.92, −1.45) for prevalence and −2.01 (95% CI −2.21, −1.82) for DALYs, respectively. The AAPC for FBT prevalence and DALYs in Japan from 1990 to 2021 was 1.067 (95% CI 1.00, 1.14) for prevalence and 1.04 (95% CI 0.98, 1.11) for DALYs, respectively. The AAPC of FBT prevalence and DALYs in South Korea from 1990 to 2021 was −0.557 (95% CI −0.63, −0.49) for prevalence and −0.43 (95% CI −0.48, −0.38) for DALYs, respectively. Overall, the global prevalence of FBT and DALYs showed a fluctuating downward trend from 1990 to 2021, with AAPC of −2.18 (95% CI −2.36, −2.00) for prevalence and −2.45 (95% CI −2.60, −2.30) for DALYs, respectively. China experienced more significant declines in FBT prevalence and DALYs from 1990 to 2021 than South Korea, but the FBT disease burden in both countries was still higher than the global average. While Japan’s burden of FBT in 2021 marked a notable increase compared to 1990, it remained considerably lower than the global mean.

### 3.3 Gender disparities in the burden of FBT in different age groups in China, Japan, South Korea, and the world

**Figure 3:**
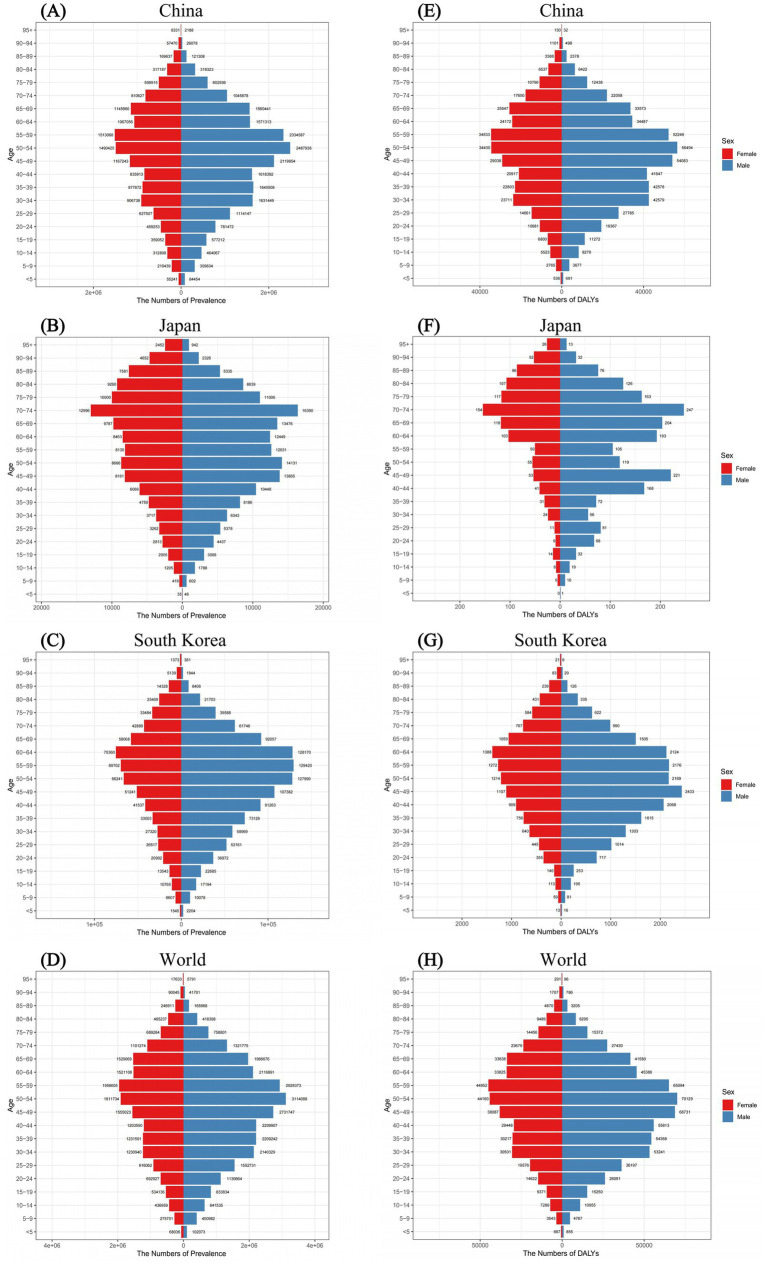
Comparison of the prevalence of FBT in males and females of different age groups for the year 2021 in China **(A)**, Japan **(B)**, South Korea **(C)**, and worldwide **(D)**. Comparison of the DALYs of FBT in males and females of different age groups for the year 2021 in China **(E)**, Japan **(F)**, South Korea **(G)**, and worldwide **(H)**.

Figure 3 illustrated the prevalence and DALYs of FBT in different age groups of males and females in China, Japan, South Korea, and the world in 2021. From the prevalence results in China, the overall disease burden of FBT concentrated in the middle-aged group, with males most affected between the ages of 50 and 54 and females between the ages of 55 and 59. Similar tendencies were observed for DALYs. In Japan, the prevalence of FBT steadily climbed among those under 54 years old, peaking in the 70–74 age bracket, before declining with advancing age. While the DALYs in Japan showed a bimodal distribution in the 70–74 and 45–49 age groups. In South Korea, the peak prevalence was observed in women aged 60–64 and men aged 55–59. The highest DALYs were found in women aged 60–64 and men aged 45–49. For both genders, the prevalence and DALYs of FBT increased with age, reaching a peak and then gradually decreased. In 2021, except for Japan where males under 85 had a higher prevalence of FBT than females, in China and South Korea, males under 80 across all age groups had a higher prevalence than females, with the trend reversing after age 80.

### 3.4 Burden of FBT in different age groups in China, Japan, South Korea, and the world in 1990 and 2021

**Figure 4:**
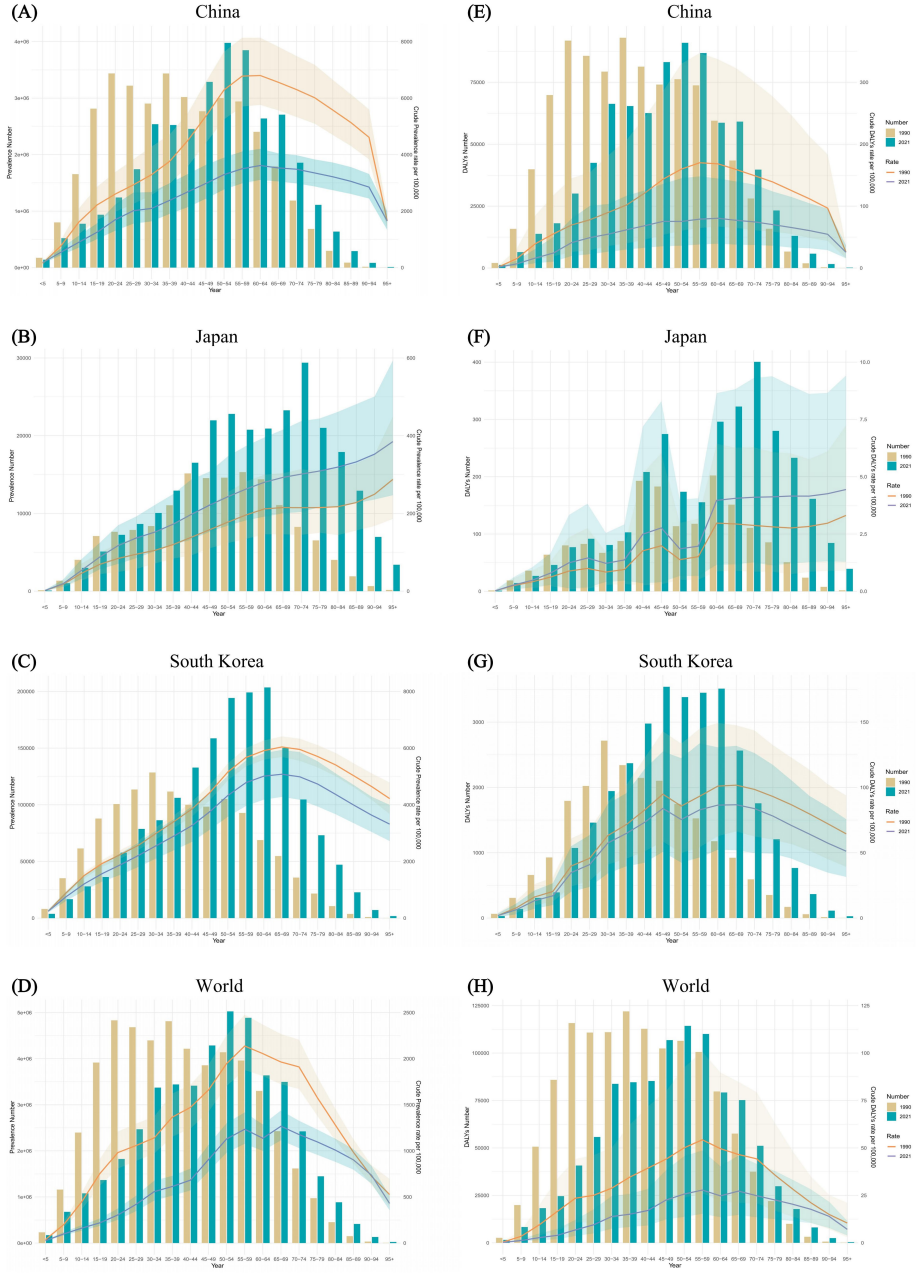
Comparison of the prevalence counts, along with their crude rates, by age group in China **(A)**, Japan **(B)**, South Korea **(C)**, and worldwide **(D)** from 1990 and 2021. Comparison of the DALYs counts, along with their crude rates, by age group in China **(E)**, Japan **(F)**, South Korea **(G)**, and worldwide **(H)** over the same period. Bar charts represent counts; lines represent crude rates.

Figure 4 presented the prevalence and DALYs of FBT across various age groups for China, Japan, South Korea, and globally in the years 1990 and 2021, accompanied by their respective crude rates. From the prevalence rate results in China, FBT prevalence escalated notably among young people, peaking in middle-aged individuals, particularly within the age group of 30–60 years old. Specifically, the crude prevalence rate (CPR) of FBT in China demonstrated a progressive increase from the 0–5 age group to the 60–64 age group, followed by a decline in the advanced age ranges. Similar trends were observed in the crude DALY rate (CDR). In 2021, the CPR for FBT in Japan showed a pronounced age-related increase, exhibiting a clear upward trajectory compared to the rates in 1990. In both 1990 and 2021, the CDR for FBT in Japan exhibited a fluctuating yet overall increasing pattern with advancing age. Both in 1990 and 2021, the CPR of FBT in South Korea showed an increasing trend from the 0–5 age group to the 65–69 age group. In 1990, the peak prevalence of FBT in South Korea was in the 30–34 age group, while in 2021, it was in the 60–64 age group. Additionally, in 2021, the 45–64 age group consistently exhibited an elevated CDR for FBT.

### 3.5 Trends in the all-age cases and ASPR and ASDALR of FBT by sex in China, Japan, South Korea, and the world from 1990 to 2021

**Figure 5:**
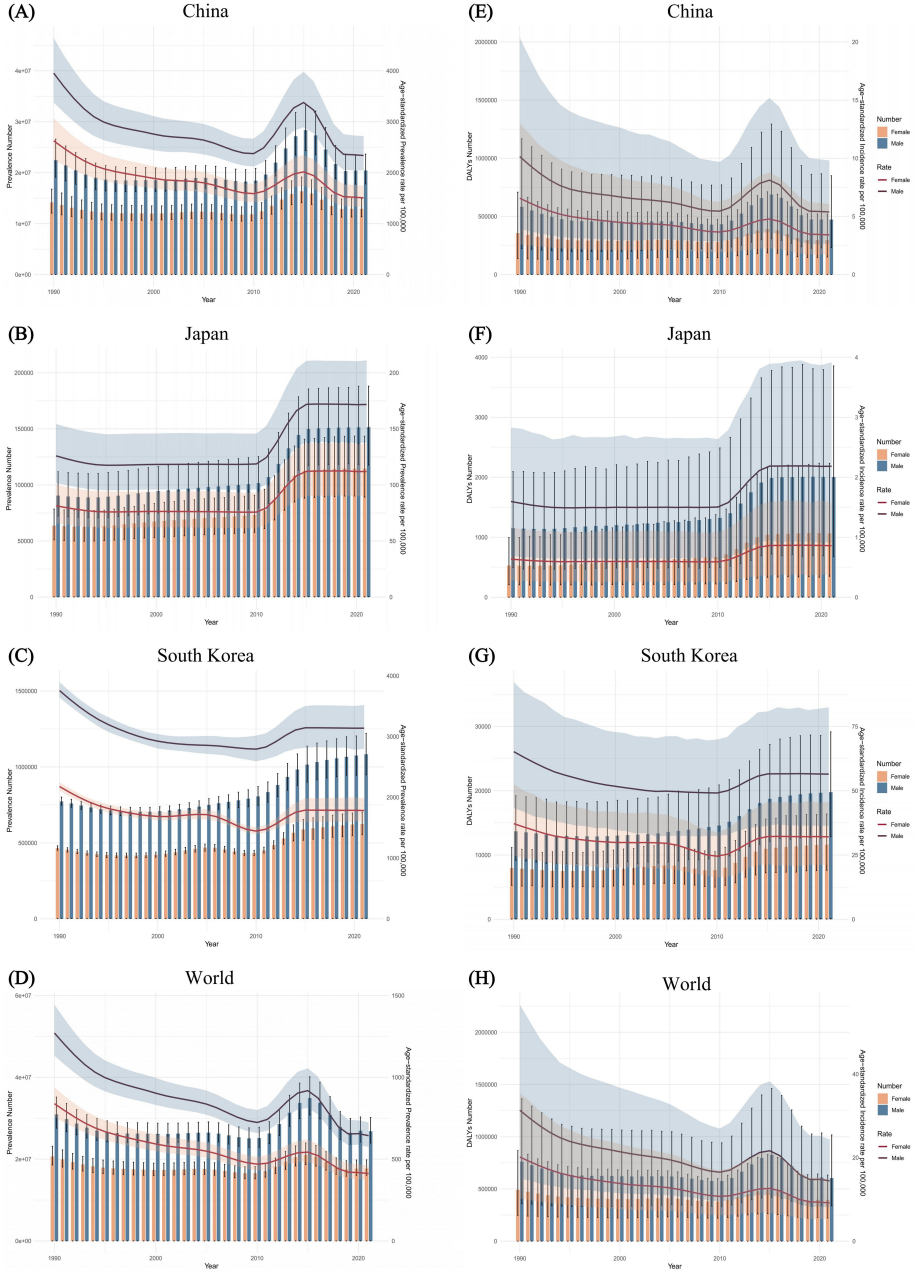
Trends in the all-age prevalence number and ASPR of FBT by sex from 1990 to 2021 in China **(A)**, Japan **(B)**, South Korea **(C)**, and worldwide **(D)**. Trends in the all-age DALYs number and ASDALR of FBT by sex for the same period in China **(E)**, Japan **(F)**, South Korea **(G)**, and worldwide **(H)**.

In China, the sex-specific, ASPR and ASDALR of FBT fluctuated over the calendar years, exhibiting an overall downward trend. The temporal trends for both sexes were similar, with the magnitude of change in males consistently greater than that in females. From 1990 to 2021, the sex-specific all-age number and age-standardized rates of FBT prevalence and DALYs in Japan increased for both sexes. In South Korea, there was a slight decline in the ASPR and ASDALR for both males and females. However, the overall number of cases and DALYs for both sexes did not decrease. Males consistently had a higher prevalence and higher DALYs of FBT than females. In 1990, the greatest disparity in FBT prevalence and DALYs rates between males and females was observed in China, South Korea, and globally, while in Japan, the largest difference was noted in 2014 (Figure 5).

### 3.6 Predictions of the disease burden of FBT in China, Japan, South Korea, and the world from 2020 to 2030

**Figure 6:**
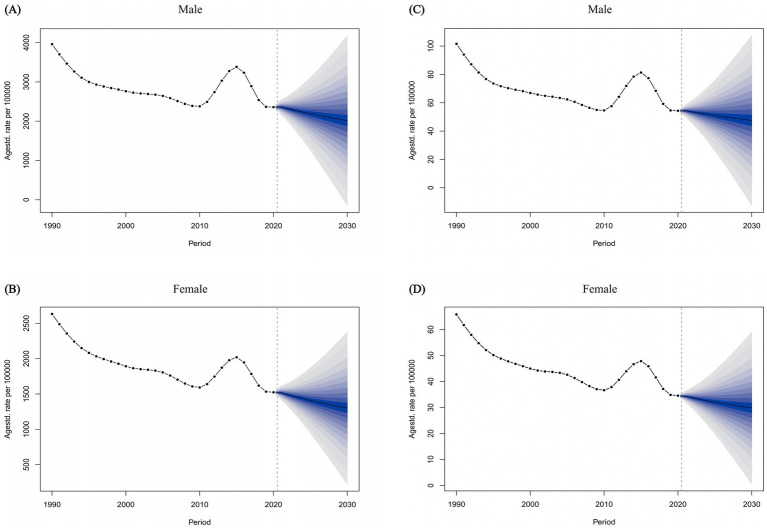
The temporal trends of the ASPR of FBT for males **(A)** and females **(B)** from 1990 to 2021 in China, with projections extending until 2030. The temporal trends of the ASDALR of FBT for males **(C)** and females **(D)** from 1990 to 2021 in China, with projections extending until 2030.

Ultimately, we employed the BAPC method to project the ASPR and ASDALR for FBT in China from 2021 to 2030 (Figure 6). Our analysis revealed that from 2021 to 2030, both the ASPR and ASDALR for FBT in China were anticipated to decrease, with a higher prevalence rate observed in males compared to females. Specifically, the ASPR for FBT among males in China was projected to decline gradually, with an estimated 2,015.64 cases by 2030, and approximately 1,301.79 cases for females. Furthermore, the ASDALR for Chinese males with FBT was expected to reduce to around 4.75 by 2030, while the ASDALR for females was projected to be about 2.98. Likewise, the ASPR and ASDALR of FBT were projected to decrease in Japan and worldwide between 2021 and 2030. In contrast, a modest increase in the disease burden was anticipated for South Korea during the same period (Supplementary Figures 1, 2).

## 4 Discussion

In this study, using data from the GBD 2021, we conducted a comprehensive assessment and comparison of the burden and temporal trends in the prevalence of FBT in China, Japan, and South Korea over the past 30 years, and further predicted the prevalence and DALYs of FBT in China by 2030. The global burden of FBT has been on a downward trend, yet the Asian region as a whole continues to experience a high disease burden of FBT. China, Japan, and South Korea are neighboring countries in Asia, each being a major economy with similar living habits. However, significant disparities exist in the FBT burden among these three nations.

Our study reveals that, despite a surge in cases around the year 2010, there has been a notable overall decline in the prevalence and DALYs of FBT in China from 1990 to 2021, which may correlate with rapid economic growth, enhanced sanitation and hygiene practices, and effective public health interventions. After being overlooked for decades, FBT was finally recognized by the World Health Organization as a neglected tropical disease in 2010 (19). This recognition heightened awareness of FBT and emphasized the importance of screening, leading to the observed increase in reported cases. Prior to this recognition, FBT was largely overlooked, resulting in limited surveillance and inadequate data collection (20). China conducted three nationwide surveys on key parasitic diseases in humans from 1988 to 1992, 2001–2004, and 2014–2016, thereby promptly understanding the epidemiological situation of various FBT in our country (21, 22). Additionally, multiple integrated prevention and control zones have been established, and significant strides have been made in the prevention and control of FBT through comprehensive strategies such as health education and drug deworming. However, the vast territory of China, characterized by its uneven geographical distribution, leads to a diversity of FBT types, and the relative lag in diagnostic and treatment technologies continues to contribute to a high burden of FBT (23). The prevalence and DALYs of FBT in Japan are significantly lower than those in China and the global average, which is attributed to Japan’s advanced economy, sophisticated multi-combination diagnostic methods, and the sharp decline in intermediate hosts of FBT (24). The burden of FBT disease in Japan is relatively small, often reported as case reports, lacking accurate epidemiological data. With globalization and rapid population movement, imported cases from neighboring countries have led to an increase in Japan’s disease burden in recent years. However, the Japanese government currently does not have a detection and control plan for FBT (24, 25). In South Korea, although the ASPR and ASDALR have slowly declined from 1990 to 2021, the absolute numbers of prevalence and DALYs remain high, indicating that South Korea needs to further strengthen its efforts in controlling FBT in the future.

Current research indicates that over the past three decades, the primary affected group for FBT in China, Japan, and South Korea has been the middle-aged population, a finding consistent with the general pattern of FBT occurrence (3). Due to the accumulation of long-term FBT infections, the prevalence of FBT increases with age, peaking at 60 years in China. Cancers stimulated by trematodes are rare in patients under 40, typically occurring in those aged 60 and above. The slight decrease in prevalence among the older adult may be attributed to the premature death of infected individuals due to cholangiocarcinoma and other related complications (26, 27). In these three countries, the age distribution peak of the FBT burden shifts toward older age groups, especially in Japan. This phenomenon is closely related to the aging population structure. Population aging is a result of rapid economic growth and the development of healthcare, affecting all three countries to varying degrees. As of 2020, the older adult population aged 65 and over accounted for 28.7% of Japan’s total population, making it one of the most aged countries globally (28). South Korea has the world’s lowest fertility rate, and its pace of aging is even faster than Japan’s. It is projected to enter a “super-aged” society around 2025, where the older adult population aged 65 and over will account for more than 20% of the total population (29). With its large population, it is expected that by 2030, the older adult population aged 65 and over in China will exceed 230 million.^3^ This indicates that FBT in the older adult is a health issue of common concern for the three countries in the future. The older adult are more susceptible to FBT due to decreased immune function, weakened resistance, and a higher likelihood of comorbidities, including tumors.

In China, Japan, and South Korea, the burden of FBT infections tends to be higher in males, particularly among those under the age of 80. This gender disparity may stem in part from differences in social engagements, dietary practices, occupational choices, and health risk awareness between males and females. Men may more frequently engage in social gatherings and might be more likely to consume unconventional or risky foods, such as raw or poorly cooked freshwater fish, potentially increasing their risk of contracting FBT. Additionally, men are more likely to work in professions like fishing and agriculture, where they may come into direct contact with sources of water contamination or handle aquatic products that could be infected (30). Moreover, differences in immune system functions and hormonal levels between genders may also affect susceptibility to parasitic infections. The interaction of these biological factors with socio-behavioral elements may lead to variations in the burden of FBT infections among different genders and age groups. However, among those aged 80 and above, the burden of FBT infections is higher in females, possibly linked to the fact that women generally have longer life expectancies than men (31). These insights highlight the importance of accounting for gender differences in public health strategies and emphasize the need for targeted preventive measures to mitigate the occurrence of FBT infections.

Recent assessments have shown an increase in human FBT diseases, primarily attributed to the exponential increase in aquaculture production (32, 33). Individuals residing in close proximity to freshwater bodies face a 2.15-fold higher risk of infection compared to those living at a distance (95% CI 1.38–3.36) (34). China, Japan, and South Korea play significant roles in the Asian aquaculture industry, with their collective output being notably substantial on a global scale. Asia represents the largest aquaculture producer, accounting for approximately 90% of global aquaculture output in 2017, with China contributing 58% of the worldwide production (35). However, the proliferation of aquaculture has precipitated ecological transformations and introduced a myriad of diseases in aquatic animals (32). In aquaculture settings, adverse environmental conditions compromise fish immune systems, allowing for easier infection by pathogens, including parasites and bacteria. Moreover, high-density farming increases interactions among fish, promoting the transmission of diseases. This is a critical factor in the transmission of FBT to humans through the consumption of infected fish (36).

Like aquaculture workers, fishermen, owing to the characteristics of their occupation, have long-term contact with water bodies and aquatic products and are more susceptible to FBT infection than the general population (37). For example, in some areas, fishermen, eager to eat the freshwater fish they catch, simply process or even eat them raw, which significantly increases the risk of infection (38). In agriculture, many farmers directly use river water as domestic or drinking water, which may expose them to water containing trematode larvae (39). The suitable temperature and humidity, and the rich vegetation on farms provide a favorable environment and ample food for FBT intermediate hosts, such as snails (40). Cats, dogs, and pigs are also important hosts of FBT and are commonly found on farms. These animals are usually allowed to move around freely and defecate at will, which may result in the contamination of the farm environment by the parasite eggs (41, 42). In addition, many farmers, in order to obtain profits from fertilizer, directly use untreated animal and human feces as organic fertilizer, which is applied to agricultural soil and ponds. This practice not only pollutes agricultural soil and water sources but also contaminates vegetables and green fodder products (43–45).

Additionally, seasonality is an important factor in studying the epidemiology of FBT (46, 47). The seasonality of FBT is not accidental but results from multiple factors. Biologically, the reproduction and activity patterns of intermediate hosts are significantly influenced by seasonality. For example, there is a strong correlation between rainfall or evapotranspiration and both snail population size and FBT infection rates in snails (48). During the rice-growing season, human irrigation activities can alter this relationship (49). This is because seasonal temperature fluctuations and the rainfall-determined availability of surface water largely influence the dynamics of snail populations (50). In terms of human activities, both dietary habits and the growth and consumption cycles of aquatic products and vegetables are strongly associated with seasonality (51, 52). Specifically, the growth of vegetables is influenced by seasonal climate, and the risk of carrying infectious metacercariae varies seasonally (53). Studies have shown that FBT infections in humans and animals exhibit distinct seasonal patterns, and the infection risk associated with vegetable consumption peaks in June and July, although the intensity of this peak varies by topography and climatic zone (54).

Due to the significant differences in natural environments and climatic conditions, diverse industry forms and regional cultures have emerged across different geographical locations globally. In the Americas, Africa, and Europe, where agriculture and animal husbandry are more developed, frequent contact with animals and plants during production activities increases the risk of FBT infection. In the field of fisheries, the Americas, Europe, the Eastern Mediterranean, and the Western Pacific regions, with their superior coastlines and marine resources, have become important fishing areas globally. In some areas of these regions, unique raw-food cultural traditions are preserved, further increasing the risk of infection. It is worth noting that, despite varying levels of development, the Americas, Africa, Europe, the Eastern Mediterranean, and the Western Pacific all face challenges related to poverty and low socioeconomic status to varying degrees (4, 55, 56). Economic conditions significantly influence the prevalence of FBT through multiple pathways. First, poverty restricts people’s access to basic health care and sanitation, becoming a major cause of parasitic infections (57, 58). In low-income areas, the lack of sanitation facilities and water pollution provide conditions for the transmission of waterborne parasites, thereby exacerbating the disease burden of FBT (59–61). In addition, the prevalence of FBT is also closely related to the prevention and control capabilities at the national level. In many low-income and middle-income countries, due to a lack of sufficient political attention and investment, there are many shortcomings in the development of laboratory detection technology, allocation of financial resources, and the construction of monitoring systems. For example, the lack of advanced laboratory techniques makes it impossible to detect FBT infections in a timely and accurate manner, leading to difficulties in identifying and controlling outbreaks; the incomplete monitoring system fails to fully grasp the epidemiological trends of FBT in the population, making control measures lack specificity (62–64). At the same time, in the realm of food safety, low-income and middle-income countries, because of insufficient regulatory capacity in food safety, experience much higher incidence and mortality rates of FBT relative to high-income countries (65). These heavy burdens of FBT, in turn, lead to significant productivity losses, thereby creating a vicious cycle of “poverty - high disease incidence - productivity loss - greater poverty.”

In recent years, with improvements in the economic environment and increased health awareness, the epidemiology of FBT diseases has shifted. In certain instances, a notable decrease in the prevalence of FBT infections has been observed, which can be attributed to factors such as socio-economic progress, stringent food inspections, enhanced sanitary conditions, health education initiatives, and the application of praziquantel (66, 67). However, the lifestyle habits and dietary customs across various regions of our country exhibit considerable diversity. With the rise in living standards, raw and specialty diets have become trendy, and with the advancement of logistics, the geographical boundaries for FBT diseases have been transcended, leading to occasional outbreaks of clustered infections (68). With the ongoing process of urbanization, the prevalence of FBT has transitioned from rural to urban areas, with a significant increase in incidence rates among urban populations compared to their rural counterparts (69). This emerging trend presents novel challenges for the prevention and control of FBT diseases in our country.

Therefore, health education targeting FBT is crucial (9, 70, 71). Key points include raising awareness about the transmission routes and risks of parasitic diseases, with a particular emphasis on avoiding undercooked fish, crustaceans, and aquatic plants. Standardizing food handling through scientific processing and storage methods to reduce the risk of parasitic contamination. Strengthening education for high-risk groups, such as fishermen and farmers, through tailored education programs to enhance their self-protection awareness. Promoting good personal hygiene practices, such as handwashing before and after meals and avoiding untreated water. Maintaining environmental hygiene by focusing on water source protection, preventing wastewater contamination of water sources, and enhancing sanitary management of aquaculture farms and slaughterhouses. Promoting health education through national health literacy campaigns and extending it to communities, schools, and households to guide the public in adopting healthy lifestyles.

However, this study has several limitations. Firstly, this study utilizes the GBD 2021 dataset for data analysis, the dataset’s approach to estimating missing data by adjusting source data may affect the accuracy of the information. Secondly, the classification of FBT is constrained by data limitations and does not clearly delineate the specific variables for each trematode species. Consequently, this study focuses solely on the overall burden trends of FBT. Lastly, the diagnostic and detection methods for FBT may evolve over time, and variations in data collection methods and tools across different eras may introduce potential biases. The GBD database was last updated in 2021, which highlights an urgent need for the most current epidemiological data from 2024.

## 5 Conclusion

In summary, we conducted a comprehensive and in-depth analysis of the burden of FBT diseases in China from 1990 to 2021, and for the first time, we have compared this with the situations in Japan and South Korea. From 1990 to 2021, the burden of FBT diseases in China significantly decreased, and it is projected to continue decreasing by 2030. However, the China’s burden remains higher than that in Japan and the global average. The burden of FBT in South Korea has slightly decreased, but further control measures are needed. Japan maintains a lower burden of FBT diseases but must remain vigilant against the recent upward trend. Control of FBT requires multi-sectoral collaboration, including the joint efforts of public health, medical, educational, and environmental sectors, to achieve the World Health Organization’s goal of controlling neglected tropical diseases by 2030. Through interdisciplinary collaboration and the support of global partners, we can more effectively address neglected tropical diseases like FBT, protecting and promoting the health of both humans and animals.

## Data Availability

The original contributions presented in the study are included in the article/Supplementary material, further inquiries can be directed to the corresponding author.

## Footnotes

^1^ https://www.who.int/publications/i/item/9789240010352

^2^ http://ghdx.healthdata.org/gbd-results-tool

^3^ www.stats.gov.cn/
