# Supplementary material for: Prevalence trend and burden of foodborne trematodiasis in China from 1990 to 2021 and its predictions until 2030: a comparative study with Japan and South Korea
Source: Front Public Health. 2025 Feb 24;13:1504218. doi: 10.3389/fpubh.2025.1504218 (PMC11891204; doi:10.3389/fpubh.2025.1504218)
Supplement: Supplementary file 1 [file Presentation_1.PDF]

Foodborne trematodiases

Clonorchiasis

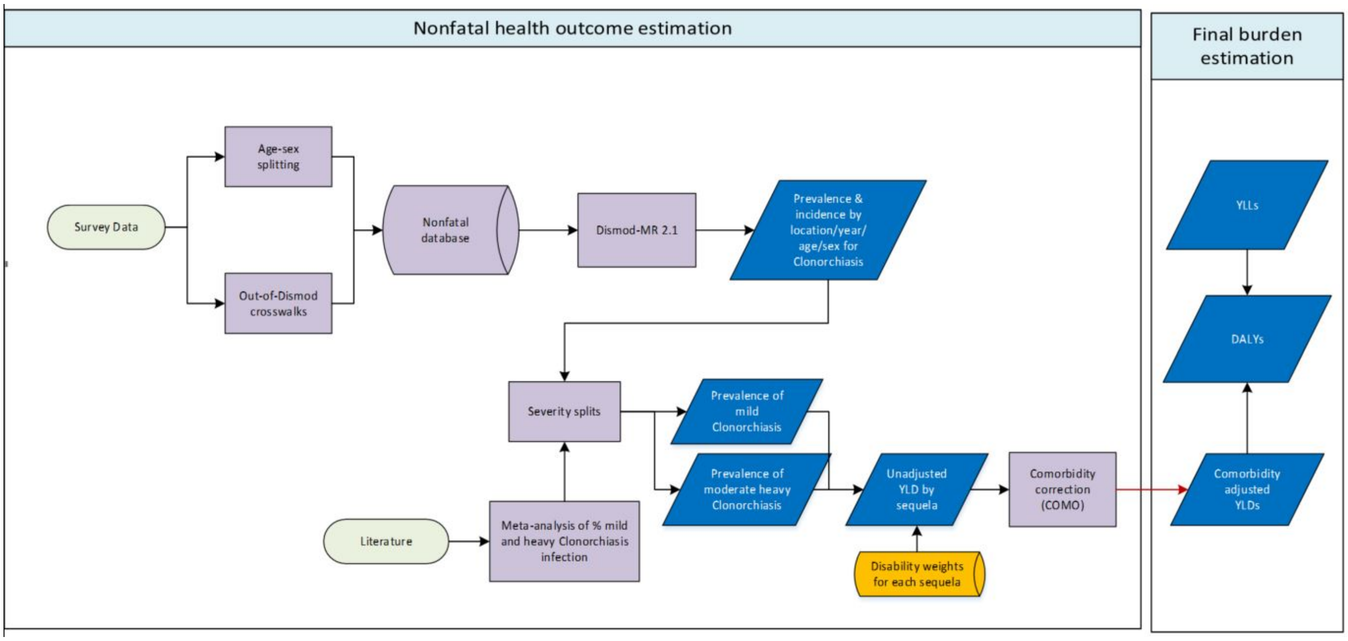

Fascioliasis

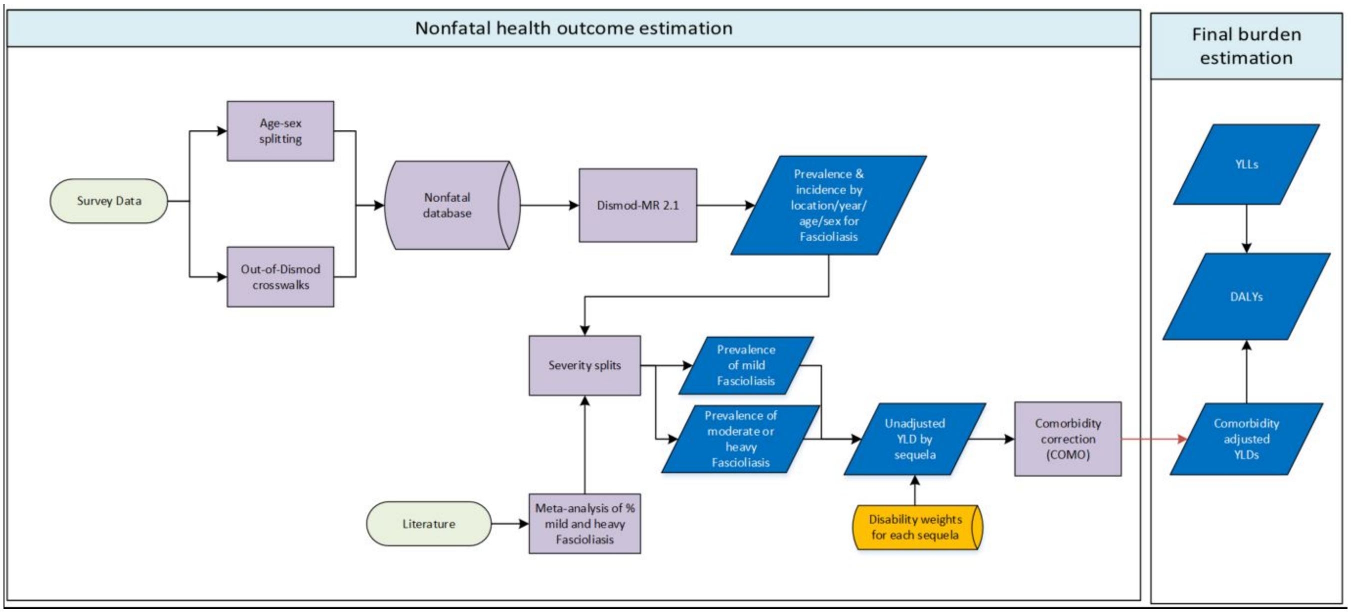

Intestinal fluke

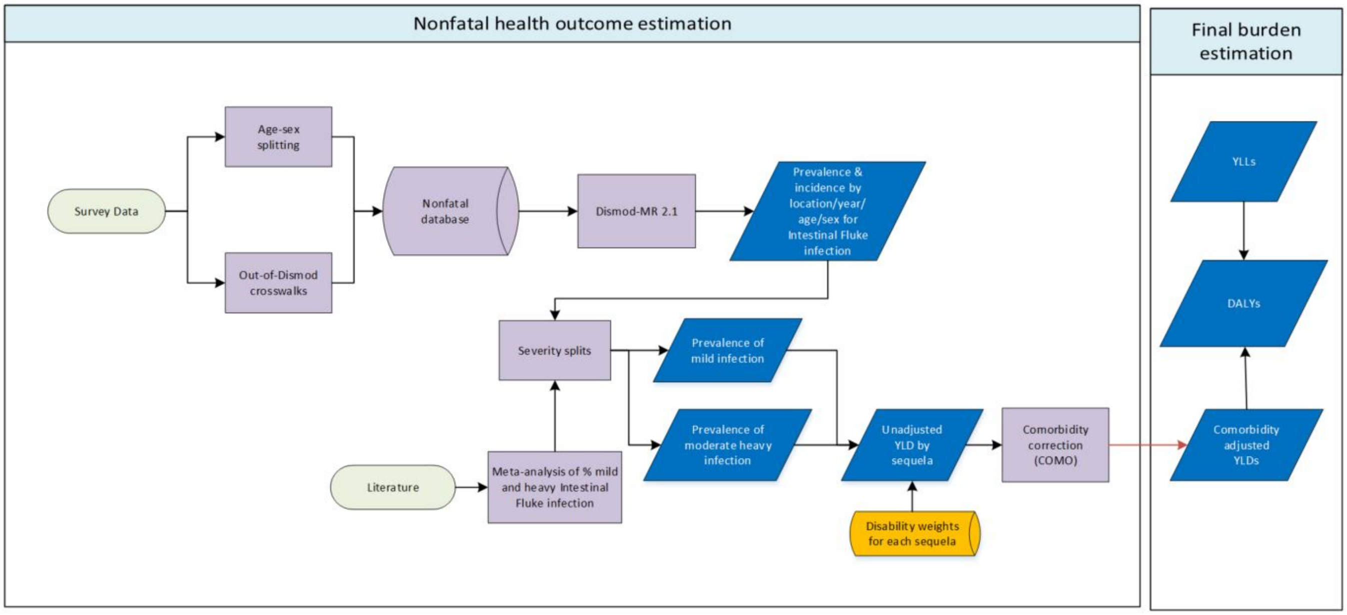

Opisthorchiasis

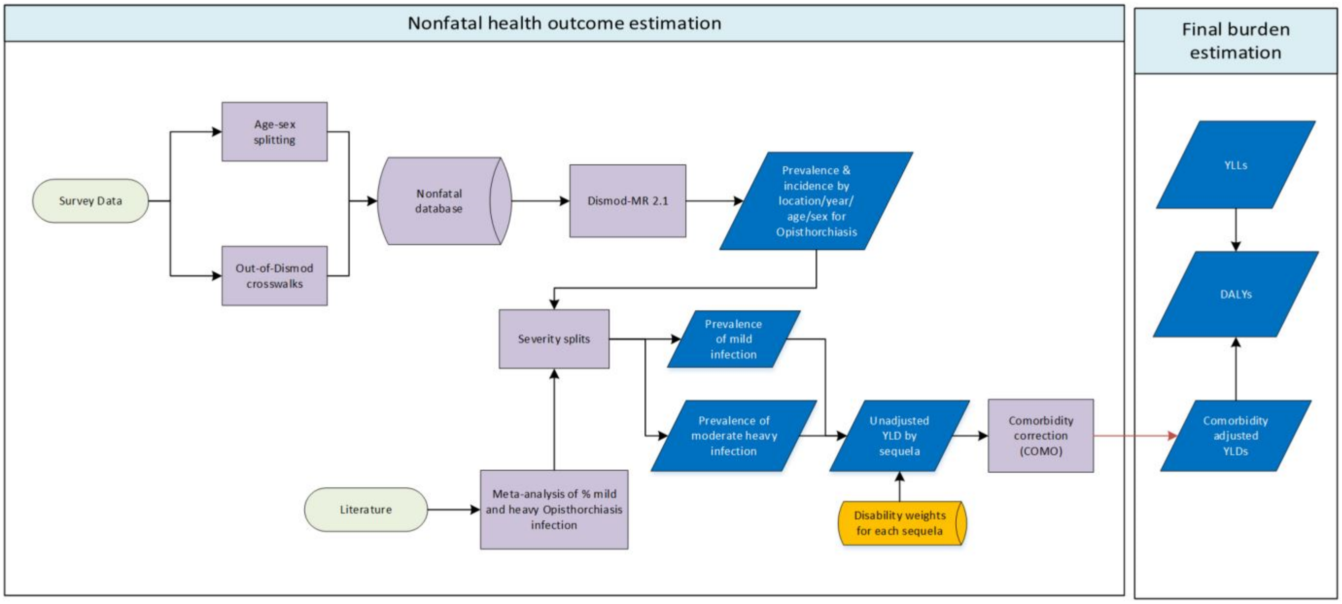

Paragonimiasis

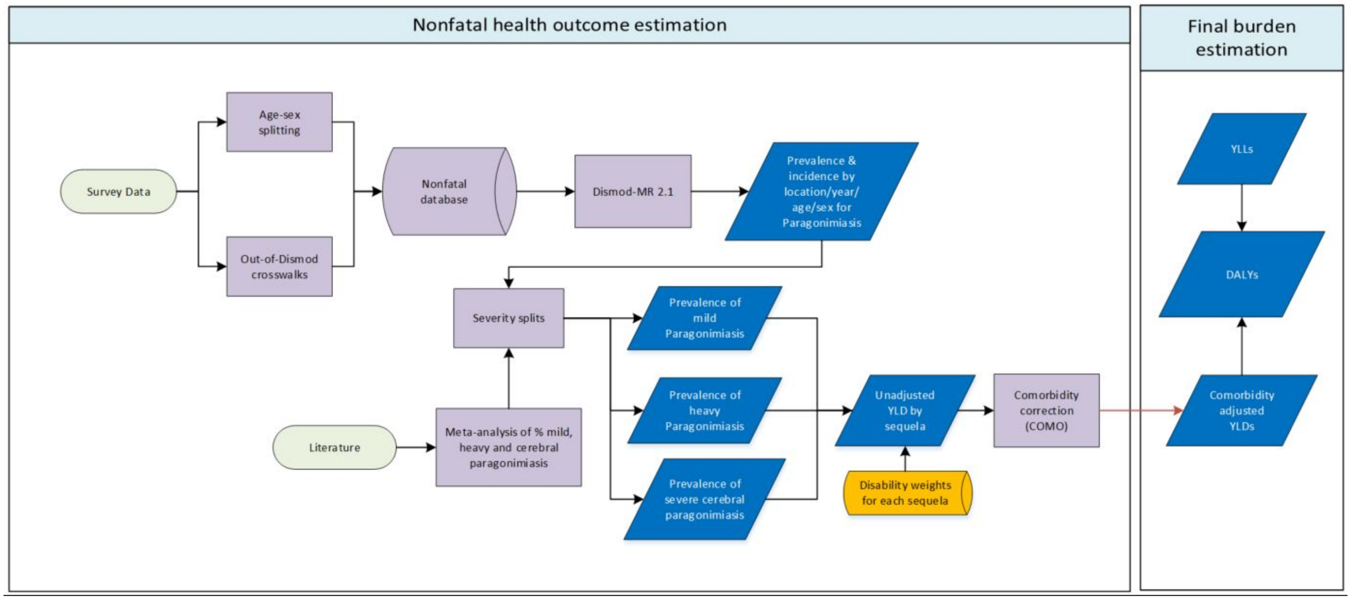

Input data and methodological summary

Case definition

The foodborne trematodiases (FBT) are a group of diseases that result from infection with parasitic worms of the class Trematoda, also known as flukes, via consumption of contaminated food. Infection of the liver, gallbladder, lungs, or brain can result in abdominal pain, chronic respiratory symptoms, neurologic symptoms including epilepsy, and cholangiocarcinoma (bile duct cancer). In the ICD-10, FBT are listed under code B66 [1]. FBT is subdivided into six types of FBT (see Table 1):

- Clonorchiasis
  Clonorchiasis is a parasitic disease that results from infection with the liver fluke *Clonorchis sinensis*, transmitted primarily via consumption of raw or undercooked fish. In addition to acute infectious symptoms, longer-term complications can result from inflammation of the liver, gallbladder, and pancreas and biliary obstruction. Clinical manifestations include abdominal pain, nausea, vomiting, weight loss, fatigue, jaundice, and cholangiocarcinoma (bile duct cancer).
- Fascioliasis
  Fascioliasis is a parasitic disease that results from infection with the liver flukes *Fasciola hepatica* or *Fasciola gigantica*, transmitted primarily via consumption of contaminated raw water plants such as watercress. Acute clinical manifestations include abdominal pain, nausea, vomiting, fever, and rash, while chronic manifestations include jaundice, hepatomegaly, and weight loss, along with inflammation of the liver, gallbladder, and/or pancreas.
- Intestinal fluke
  Intestinal flukes are a diverse set of parasites including *Fasciolopsis buski, Metagonimus yokogawai, Heterophyes heterophyes, Echinostoma* species, and others, which can cause disease after infection of the intestinal tract. They are most commonly acquired via consumption of water plants, fish, and/or crustaceans. Many infected individuals are asymptomatic, but clinical manifestations can include abdominal pain, diarrhea, vomiting, or weight loss.
- Opisthorchiasis
  Opisthorchiasis is a parasitic disease that results from infection with the liver flukes *Opisthorchis felineus* or *Opisthorchis viverrini*, transmitted primarily via consumption of raw or undercooked fish. In addition to acute infectious symptoms, longer-term complications can result from inflammation of the liver,

gallbladder, and pancreas and biliary obstruction. Clinical manifestations include abdominal pain, nausea, vomiting, weight loss, fatigue, jaundice, and cholangiocarcinoma (bile duct cancer).

- Paragonimiasis (normal and cerebral infections)
  Paragonimiasis is a parasitic disease that results from infection with lung flukes of the genus *Paragonimus*, most commonly *Paragonimus westermani*, transmitted via consumption of contaminated food - most commonly raw or undercooked crabs, crayfish, or snails. Acute infection can result in fever, abdominal pain, rash, chest pain, and cough; late infection can cause hemoptysis (cough with bloody sputum). Less common clinical manifestations result from spread of the parasite outside of the lungs, including to the central nervous system (causing meningitis or encephalitis, resulting in headache, fever, vomiting, and/or seizures), the intestines (causing nausea, vomiting and/or diarrhea), the kidneys (causing bloody urine), or the skin (causing skin nodules).

Table 1. Subtypes of FBT

	Species of FBT	Category	Carcinogen
1	Clonorchiasis	Liver fluke	Associated with cholangiocarcinoma
2	Opisthorchiasis<br><br>(*O viverrini & O felineus*)	Liver fluke	Associated with cholangiocarcinoma<br><br>(*O viverrini*)
3	Fascioliasis	Liver fluke	No available evidence
4	Intestinal flukes (*Fasciolopsis buski, Metagonimus yokogawai, Heterophyes heterophyes, Echinostoma species,* and others)	Intestinal fluke	No available evidence
5	Paragonimiasis	Lung fluke	

Case definitions used for estimation of non-fatal health burden of FBTs

**Quantity of interest**	**Reference or alternative**	**Definition**
Clonorchiasis	Reference	Prevalence of Clonorchiasis fluke infections identified by the presence of eggs in microscopic examination of stool or serological tests.
Opisthorchiasis	Reference	Prevalence of Opisthorchiasis fluke infections identified by the presence of eggs in microscopic examination of stool.
Fascioliasis	Reference	Prevalence of Fascioliasis fluke infections identified by the presence of eggs in microscopic examination of stool or serological tests.
Intestinal fluke	Reference	Prevalence of Intestinal fluke infections identified by the presence of either adult worms or eggs in the examination of stool.
Normal and cerebral Paragonimiasis	Reference	Prevalence of Paragonimiasis fluke infections identified by the presence of eggs in microscopic examination of stool or serological tests.

Thresholds for heavy infection and duration by species of FBT

The majority of people infected with FBTs are asymptomatic. When symptoms do occur, they are often non-specific. Among the clinical symptomatic group, severity is associated with worm burden, typically measured by faecal egg counts, and the duration of infection. The thresholds for heavy infection and duration by species of FBT are shown in Table 2. The clinical presentation of FBT depends on the target organs (liver, lung, or intestines). Clonorchiasis and opisthorchiasis patients may suffer from loss of appetite, fullness, indigestion, diarrhoea, pain in the right upper quadrant, lassitude, weight loss, ascites, and oedema.[2, 3] Cholangitis, obstructive jaundice, intra-abdominal mass, cholecystitis, and gallbladder or intrahepatic stones may occur as complications.[3, 4]

Table 2. Thresholds for heavy infection and duration by species of FBT

	Species of FBT	Case thresholds for heavy infection	Duration
1	Clonorchiasis	10,000 eggs per g of faeces	lifelong
2	Opisthorchiasis	10,000 eggs per g of faeces	lifelong
3	Fascioliasis	1,000 eggs per g of faeces	lifelong
4	Intestinal fluke	1,000 eggs per g of faeces	lifelong
5	Paragonimiasis	100 eggs per 5 ml sputum	lifelong
6	Cerebral paragonimiasis	Any infection of the brain with flukes and/or eggs of *Paragonimus* spp.	lifelong

Input data

Table 3: Source counts

Measure	Total sources	Countries with data
All measures	57	18
Prevalence	56	18
Proportion	1	0

Model inputs

For GBD 2010, the data came from an expert group analysis, which used the results of a systematic literature review performed by Furst and colleagues as a starting point.[5] Furst and colleagues searched PubMed,

WHOLIS, FAOBIB, Embase, CAB Abstracts, Literatura Latino Americana e do Caribe em Ciências de Saùde (LILACS), ISI Web of Science, BIOSIS preview, Science Direct, African Journals OnLine (AJOL), and the System for Information on Grey Literature in Europe (SIGLE), period Jan 1, 1980, to Dec 31, 2008. The initial number of studies identified through the literature review was ~34,000 references. The literature review included extracted data from 181 studies. For GBD 2013 and GBD 2015, the search strategy was replicated to capture epidemiological studies published between 2008 and 2015.

Input data for the assessment of the total national number of infected people

Only studies that used countrywide surveys to estimate the national prevalence rates were included (or for China, province-wide surveys). We included only national studies because FBT shows a highly focal spatial distribution and local cross-sectional surveys would profoundly under- or overestimate true national prevalence. Infection is highly related to food habits, and there are highly varying differences between national and subnational prevalence rates. This search was last updated for GBD 2015; the final dataset contained 29 prevalence studies from 17 countries. We used raw data from the selected studies as input for DisMod-MR.

Prevalence of intestinal fluke infection

Intestinal fluke infections can be caused by several different pathogens, such as *Metagonimus* spp., *Echinostoma* spp., and *Neodiplostomatidae*.[6] When assessing the prevalence of intestinal fluke infection, we added the identified prevalence for each parasite species in order to obtain the overall prevalence of intestinal fluke infections. This approach may lead to a certain overestimation of the true prevalence, because people may be co-infected with more than one intestinal fluke species. There is not sufficient evidence about the proportion of co-infections to effectively account for this in our modelling process, but the resulting overestimation of the true prevalence may be offset by the assumptions made in our modelling approach and the many challenges in generating the underlying epidemiological parameters (eg, diagnostic inaccuracy in the detection of infections with the more than 50 intestinal fluke species). Also of note, the transmission sources of intestinal fluke infections are species-specific and therefore vary. For instance, *Fasciolopsis buski* is usually transmitted by eating raw water plants with the infective parasite stage attached to the water plants, whereas *Neodiplostomatidae* are transmitted by eating undercooked and infested frogs, snakes, and tadpoles. Because of these different transmission pathways, the rate of co-infection might in fact be smaller than expected.

Input data to differentiate between asymptomatic and heavy infections

We estimated the proportion of heavily infected among all infected in all available national and regional cross-sectional surveys. It is expected that heavy infection increases with age, and there are data available on heavy infection by age group. We therefore decided to include age-dependent rates of heavy infection for clonorchiasis, opisthorchiasis, and intestinal fluke infection. For (cerebral) paragonimiasis and fascioliasis there were not sufficient age-dependent data on high-intensity FBT infection for this approach, and we therefore used an estimate of the rate of heavy infection that did not vary by age.

Data pre-processing

We used a MR-BRT (meta-regression—Bayesian, regularised, trimmed) model with our sex-specific data to derive an estimate of the ratio of the male prevalence of all-species FBT infection to female prevalence of all-species FBT infection to split non-sex-specific data. Then, a DisMod-MR 2.1 Bayesian meta-regression model using the age-specific input data was run to derive an age pattern to apply to split the all-age data.

Table 4: MR-BRT crosswalk adjustment factors for all-species FBT infection

Data input	Reference or alternative case definition	Gamma	Beta Coefficient, Log (95% CI)*	Adjustment factor**
Female data	Ref	0.82	---	---
Male data	Alt		0.48 (-1.16 – 2.12)	1.62

**MR-BRT crosswalk adjustments can be interpreted as the factor the alternative case definition is adjusted by to reflect what it would have been had it been measured using the reference case definition. If the log/logit beta coefficient is negative, then the alternative is adjusted up to the reference. If the log/logit beta coefficient is positive, then the alternative is adjusted down to the reference.*

***The adjustment factor column is the exponentiated beta coefficient. For log beta coefficients, this is the relative rate between the two case definitions. For logit beta coefficients, this is the relative odds between the two case definitions.*

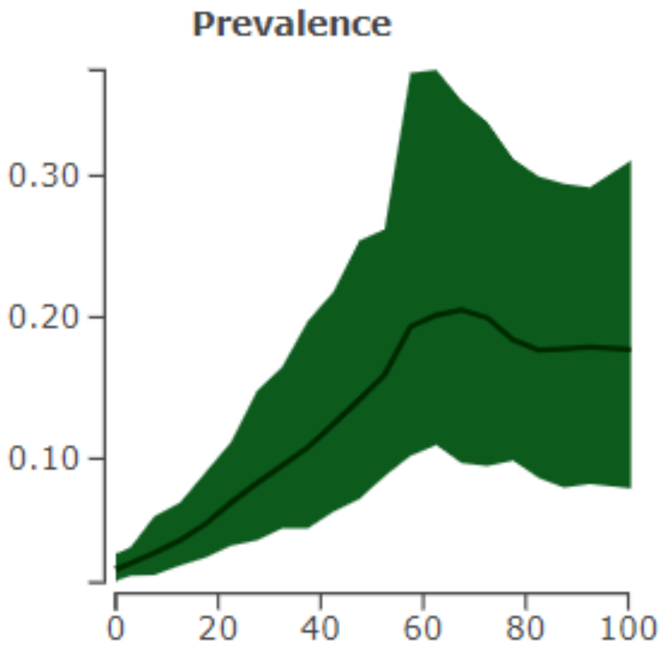

Figure 1: Global age pattern for all-species FBT infection used to split all-age data into age-specific datapoints for further modelling.

Modelling strategy

We used a three-step process for the disease modelling of FBT. In the first step we used DisMod-MR 2.0 to estimate the prevalence of FBT by age, sex, year, and country. In the second we differentiated between asymptomatic and heavy infections. MetaXL (a meta-analysis add-in for Microsoft Excel) was used to estimate the proportion of heavily infected among all infected by age group for clonorchiasis, opisthorchiasis, and intestinal fluke infection (see Table 4 and 5). These proportions were used to estimate the prevalence of heavy FBT infection. The third step consisted of deselecting countries that have no autochthonous case reports of FBTs.

Table 5. Percentage of high-intensity infection by age group and type of FBT (based on eight FBT prevalence studies)

Age category	Clonorchiasis			Opisthorchiasis			Intestinal fluke infection		
	Mean	Low	High	Mean	Low	High	Mean	Low	High
0-9	30%	17%	44%	10%	0%	29%	8%	3%	14%
10-19	15%	0%	43%	15%	0%	69%	11%	8%	14%
20-29	18%	10%	29%	16%	0%	52%	18%	15%	21%
30-39	17%	5%	34%	21%	0%	56%	22%	17%	28%
40-49	22%	13%	32%	28%	1%	68%	22%	13%	32%
50-59	18%	0%	49%	29%	0%	75%	17%	9%	28%
60+	32%	18%	47%	25%	0%	64%	15%	8%	23%

Table 6. Percentage of high-intensity infection by type of FBT (based on four FBT prevalence studies)

Type of FBT	Mean	Low	High
Paragonimiasis	23%	0%	59%
Fascioliasis	19%	3%	41%

Cerebral paragonimiasis

It was assumed that 0.8% of paragonimiasis cases have cerebral involvement. This proportion was used to estimate the prevalence of cerebral paragonimiasis. This proportion is based on one study. The data are from Oh SJ. The rate of cerebral involvement in paragonimiasis: an epidemiologic study. *Jpn J Parasitol* 1969;18:211-14. The study was performed in Paju, South Korea. This is an area with 6,738 inhabitants, and according to the survey, it was estimated that 29.6% of all individuals would react to intradermal test (an immunological reaction indicating previous or current contact with the parasite). 25% of all "positive reactors" may have eggs in their sputum (active infection with the parasite currently present in the human host). If these rates are applied to the community as a whole, the number of patients with active paragonimiasis would be at least 498 (=6,738*0.296*0.250). Furthermore, four cases of cerebral paragonimiasis were found in this community. Therefore, four out of 498 individuals with active paragonimus infection suffered from cerebral infection (=0.80%; 95% confidence interval 0.019%–1.587%).

Severity splits and disability weights

For GBD 2021, FBT was not split into health states with different severities, except for paragonimiasis. The table below shows the GBD 2021 disability weights that were used to calculate the burden of FBT in years lived with disability (YLDs).

Table 7. Disability weights that were used to calculate FBT YLDs

Sequelae	Severity description	Health state name	Disability weight

Asymptomatic clonorchiasis	Clonorchiasis, currently without symptoms	N/A	0.000 (0.000–0.000)
Heavy clonorchiasis	Abdominal pain and nausea reported as moderate	Abdominopelvic problem, moderate	0.114 (0.078–0.159)
Asymptomatic opisthorchiasis	Opisthorchiasis, currently without symptoms	N/A	0.000 (0.000–0.000)
Heavy opisthorchiasis	Abdominal pain and nausea reported as moderate	Abdominopelvic problem, moderate	0.114 (0.078–0.159)
Asymptomatic fascioliasis	Fascioliasis, currently without symptoms	N/A	0.000 (0.000–0.000)
Heavy fascioliasis	Abdominal pain and nausea reported as moderate	Abdominopelvic problem, moderate	0.114 (0.078–0.159)
Asymptomatic intestinal fluke infection	Intestinal fluke infection, currently without symptoms	N/A	0.000 (0.000–0.000)
Heavy intestinal fluke infection	Abdominal pain and nausea reported as moderate	Abdominopelvic problem, moderate	0.114 (0.078–0.159)
Mild paragonimiasis due to foodborne trematodiases	COPD and other chronic respiratory problems, mild	Has cough and shortness of breath after heavy physical activity, but is able to walk long distances and climb stairs.	0.019 (0.011-0.033)
Moderate paragonimiasis due to foodborne trematodiases	COPD and other chronic respiratory problems, moderate	Has cough, wheezing and shortness of breath, even after light physical activity. The person feels tired and can walk only short distances or climb only a few stairs.	0.225 (0.153-0.31)
Severe paragonimiasis due to foodborne trematodiases	COPD and other chronic respiratory problems, severe	Has cough, wheezing and shortness of breath all the time. The person has great difficulty walking even short distances or climbing any stairs, feels tired when at rest, and is anxious.	0.408 (0.273-0.556)
Cerebral paragonimiasis	Epilepsy	(combined DW)	--

Note. N/A: not applicable

Changes from GBD 2019 to GBD 2021

There were no major changes to our modelling approach between GBD 2019 and GBD 2021.
We did not apply any adjustments for the COVID-19 pandemic to foodborne trematodiases due to a lack of available data quantifying the impacts of the pandemic on NTD epidemiology.

References

1. WHO. *International Statistical Classification of Diseases and Related Health Problems. 10th Revision. Version for 2007.* 2007 [cited 2009 October 14, 2009]; Available from: http://apps.who.int/classifications/apps/icd/icd10online/.
2. Rim, H.J., *Clonorchiasis: an update.* J Helminthol, 2005. **79**(3): p. 269-81.
3. Pungpak, S., et al., *Clinical features in severe opisthorchiasis viverrini.* Southeast Asian J Trop Med Public Health, 1985. **16**(3): p. 405-9.
4. Rim, H.J., *The current pathobiology and chemotherapy of clonorchiasis.* Korean J Parasitol, 1986. **24**(Suppl.): p. 1-141.
5. Furst, T., J. Keiser, and J. Utzinger, *Global burden of human food-borne trematodiasis: a systematic review and meta-analysis.* Lancet Infect Dis, 2012. **12**(3): p. 210-21.
6. Furst, T., et al., *Manifestation, diagnosis, and management of foodborne trematodiasis.* BMJ, 2012. **344**: p. e4093.
