# Supplementary material for: Prevalence trend and burden of foodborne trematodiasis in China from 1990 to 2021 and its predictions until 2030: a comparative study with Japan and South Korea
Source: Front Public Health. 2025 Feb 24;13:1504218. doi: 10.3389/fpubh.2025.1504218 (PMC11891204; doi:10.3389/fpubh.2025.1504218)
Supplement: Supplementary file 3 [file Data_Sheet_2.docx]

**Supplementary Figures**

**
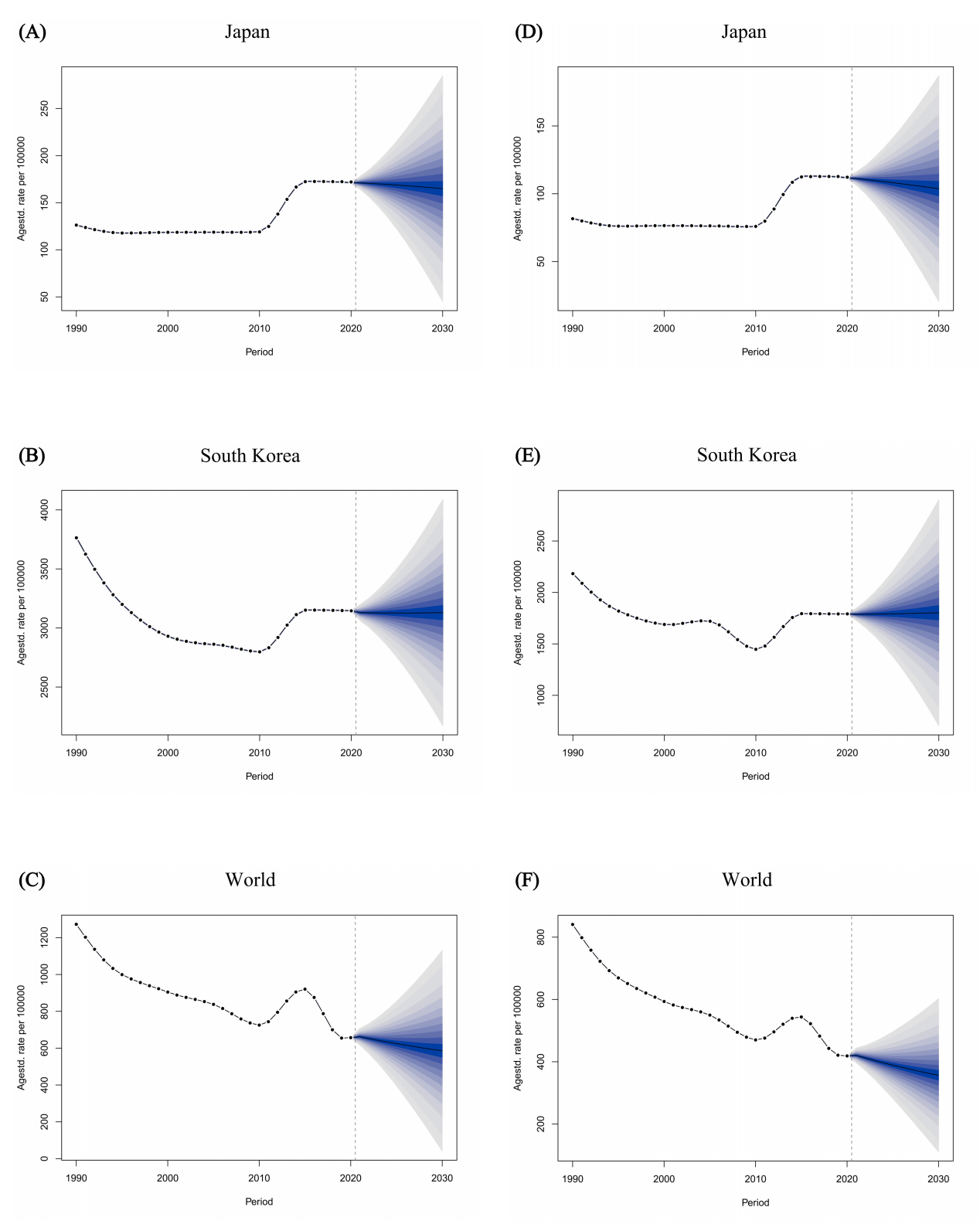
**

**Supplementary Figure 1** The temporal trends of the ASPR of FBT for males from 1990 to 2021 in Japan (Panel A), South Korea (Panel B), and worldwide(Panel C), with projections extending until 2030. The temporal trends of the ASPR of FBT for females from 1990 to 2021 in Japan (Panel D), South Korea (Panel E), and worldwide(Panel F), with projections extending until 2030.


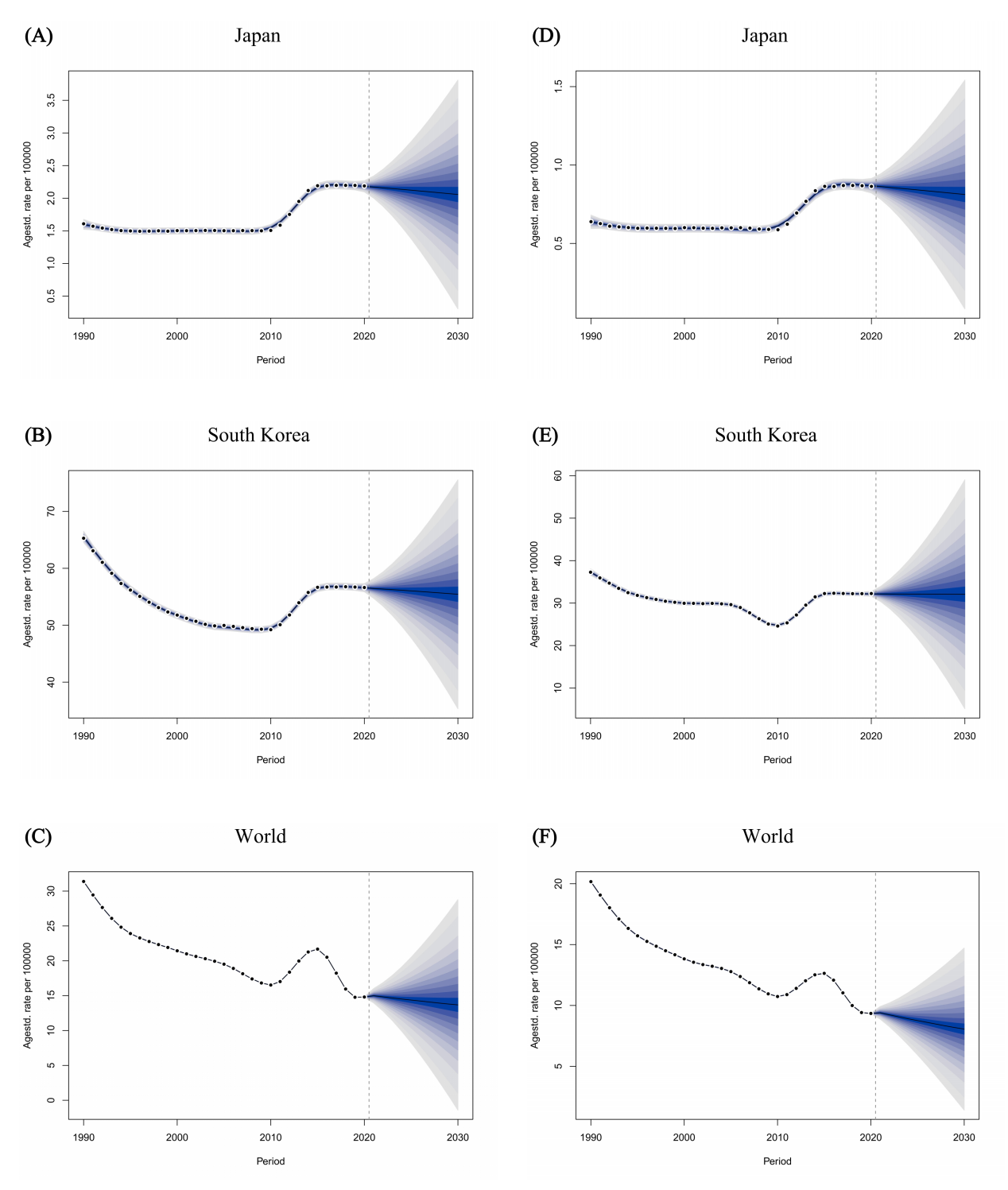


**Supplementary Figure 2** The temporal trends of the ASDR of FBT for males from 1990 to 2021 in Japan (Panel A), South Korea (Panel B), and worldwide(Panel C), with projections extending until 2030. The temporal trends of the ASDR of FBT for females from 1990 to 2021 in Japan (Panel D), South Korea (Panel E), and worldwide(Panel F), with projections extending until 2030.
